# A case series evaluating the impact of Hepatitis C eradication using direct acting antivirals on primary biliary cholangitis-associated autoimmunity

**DOI:** 10.1186/s12876-018-0826-7

**Published:** 2018-06-25

**Authors:** Henry H. Nguyen, Abdullah Khathlan, Marvin J. Fritzler, Mark G. Swain

**Affiliations:** 1 Division of Gastroenterology and Hepatology, University of Calgary Liver Unit, Calgary, Canada; 2 University of Calgary, Calgary, AB Canada; 3 Teaching Research & Wellness Centre, 3280 Hospital Drive NW, Calgary, AB T2N 4N1 Canada

**Keywords:** Primary biliary cholangitis, Hepatitis C virus, Autoimmunity, Autoantibodies

## Abstract

**Background:**

Chronic Hepatitis C Virus (HCV) infection has been commonly linked to the development of autoimmunity, in part through activation of B cells. B cells are also postulated to play a pathogenic role in the autoimmune liver disease Primary Biliary Cholangitis (PBC). Patients with concurrent PBC and HCV infection carry an increased risk of more progressive disease, although the mechanism underlying this effect is poorly understood. Utilizing a case series of patients with concurrent PBC and HCV, the aim of this study was to evaluate for the potential impact of HCV eradication upon autoimmunity/autoantibody production.

**Case presentation:**

A case series evaluating three patients with co-existing PBC-HCV infection receiving non-interferon based HCV treatments with direct-acting antivirals (DAA). One of three patient received Ursodeoxycholic acid (UDCA; 13 mg/kg/day) during the treatment period. Sustained virological response (SVR) to DAA’s was assessed using a HCV Quantitative Nucleic Acid Test (Abbott). Autoantibodies associated with autoimmune liver diseases (including PBC) and liver biochemistry, were measured before, during and after DAA treatment (Mitogen Advanced Diagnostics Laboratory, Calgary, Canada).

All patients achieved an SVR, as determined by negative HCV RNA test 12 weeks post-DAA therapy. Titres of anti-mitochondrial antibodies (AMA-M2), anti- branched-chain 2-oxo-acid dehydrogenase complex and 2-oxo glutarate dehydrogenase complex (anti-3E-BPO), and anti- tripartite motif-containing protein 21 (TRIM21/Ro52) remained unchanged, despite successful HCV eradication. Two of the three patients exhibited a transient decrease in some autoantibody titres during DAA treatment, but these returned to baseline levels post-DAA therapy.

**Conclusions:**

Within the limitations of a case series, our results suggest that HCV co-infection may not be a significant driver of PBC-related autoimmunity/autoantibody production. However, a larger n-value is required to truly assess for the effect of HCV eradication on autoantibody production.

## Background

The effects of HCV infection can extend beyond the liver. Specifically, HCV has been implicated as driving a number of extrahepatic manifestations that are characterized by autoimmunity including mixed cryoglobulinemia, glomerulonephritis, thyroid disease, Sjogren’s syndrome (SS), systemic sclerosis (SSc), and autoimmune liver disease [1, 2]. These associations suggest a close interplay between HCV and the host immune response, with HCV augmenting immune responses. How HCV infection drives autoimmunity remains unclear; however, HCV appears to be capable of directly and indirectly augmenting B cell responses [1, 3]. In general, HCV-associated autoimmune manifestations are thought to be mediated, at least in part, by autoantibodies. Antinuclear antibody (ANA), anti-smooth muscle antibody (ASMA), anti-liver kidney microsome type 1 (anti-LKM1), and anti-mitochondrial antibody (AMA) have all been previously identified in the sera of patients with chronic HCV [1, 4, 5]. Moreover, the presence of ANA, ASMA, and anti-LKM1 in HCV patients have been associated with clinical and biochemical evidence suggestive of increased liver injury [1, 6, 7]. Given the potential interactions between HCV, B cell activation and autoantibody production, defining the potential impact of HCV on coexisting autoimmune disease is of interest.

Although not common, patients with primary biliary cholangitis (PBC) and concurrent HCV infection (PBC-HCV) have been previously described [8, 9]. PBC-HCV patients appear to have more aggressive liver disease, as demonstrated by an increased risk of developing cirrhosis [8] and hepatocellular cancer (HCC), compared to patients with HCV alone [9]. Despite this increased risk, avoidance of treatment with interferon based regimens in PBC-HCV patients has been recommended due to the potential (and documented) risk of immune stimulation with drugs such as interferon exacerbating PBC [10]. With the increasing availability of direct-acting antivirals (DAA) this theoretical risk can now be circumvented. Inhibiting and/or eradicating HCV viral replication with DAA’s would remove HCV-specific stimulation of host immunity, thereby allowing for the determination of the true impact of HCV replication on driving underlying autoimmune processes; including autoantibody production associated with PBC.

Therefore, we treated three PBC-HCV patients with DAA’s and determined whether HCV replication suppression and eradication impacted PBC-related autoimmunity, as reflected by treatment-induced declines in serum levels of a number of autoantibodies associated with the diagnosis and clinical progression of PBC.

## Case presentations

Three patients with coexisting PBC and HCV were treated with interferon free DAA regimens between the years 2014 and 2015. All three patients were followed in the University of Calgary Liver Unit (UCLU) (Calgary, Alberta, Canada) which is a tertiary referral center serving Southern Alberta (~ 1.5 million people). HCV viral loads were determined before, during and after DAA treatment (ledipasvir + sofosbuvir (Harvoni®) for genotype 1a infected patients, and sofosbuvir + ribavirin for the genotype 4 infected patient) using a using Hepatitis C Virus Quantitative Nucleic Acid Test (HCV NAT; Abbott GmbH & Co., Wiesbaden, HE, Germany) performed by the Alberta Provincial Laboratory (Calgary AB, Canada). HCV-NAT were assessed at either 12 or 24 weeks post-HCV treatment to define Sustained Virologic Response (SVR). A spectrum of auto-antibodies reported to occur in the context of PBC and/or autoimmune liver diseases in general, were measured in serum samples obtained before, during and after DAA therapy. Autoantibodies targeting the following antigens were assessed: mitochondrial antibodies M2 (AMA-M2), branched-chain 2-oxo-acid dehydrogenase complex and 2-oxo glutarate dehydrogenase complex (3E-BPO), tripartite motif-containing protein 21 (TRIM21/Ro52), glycoprotein 210 (gp210), sp100 nuclear antigen (sp100), soluble liver antigen (SLA), promyelocytic leukemia cell antigen (PML), and liver cytosolic-1 antigen (LC-1). These autoantibodies were analyzed using a Line Immunoassay (Mitogen Advanced Diagnostic Laboratory, Calgary, AB Canada). Liver stiffness was measured using a Fibroscan® (carried out by experienced operators) as an indirect measure of the degree of liver fibrosis. Comparison of the patients autoantibody profiles and serum biochemistry were made against established guidelines by international liver associations for the diagnosis of PBC [11, 12].

Table 1 summarizes the patient demographics along with respective autoantibody, serological, and virological profiles. On initial assessment at the UCLU, Patient 1 tested positive for AMA in the setting of a normal serum Alkaline Phosphatase (AP) levels. Diagnostic criteria for PBC, which requires concurrent elevation in serum AP levels and a positive AMA (if no liver biopsy is performed), was therefore not met. However, Patient 1 subsequently fulfilled the diagnostic criteria for PBC with the development of cholestasis (i.e. elevated serum AP level). Treatment with Ursodeoxycholic acid (UDCA; 13 mg/kg/day) was initiated at that time and continued throughout the concurrent treatment period for HCV with DAA. Serum AP normalized with UDCA therapy and remained normal throughout the DAA treatment period.

**Table 1:** Characteristics of patients with positive anti-mitochondrial antibody and Hepatitis C Virus infection

Characteristics	Patient 1	Patient 2	Patient 3
Demographics			
Age, yrs	67	63	64
Sex, M/F	M	F	F
Anti-Mitochondrial Antibody			
Titer by IF at diagnosis	1:40	1:160	1:160
Liver Enzymes at diagnosis			
AP (N<145 IU/L)	67	134	71
GGT (N<35)	130	330	41
Hepatitis C Virus			
Duration HCV+ status, yrs	19	19	15
Genotype	1a	1a	4
Treatment Naive	Yes	No	Yes
Treatment Regimen	LDV + SOF	LDV + SOF	SOF +RBV
RNA NAT, weeks post tx	<12 IU/ml (12)	ND (24)	ND (24)
Autoantibody Profile (Mitogen)	AMA-M2+, Anti-3E-BPO+	Anti-3E-BPO+, Anti-Ro52+	AMA-M2+, Anti-3E-BPO+
Fibroscan (fPa)			
Pre-treatment with DAA	37.4	15.1	14.1
Post-treatment with DAA	19.8	12.3	No data
Ursodeoxycholic acid treatment	Yes	No (discontinued)	No

*Abbreviations*: *AP* alkaline phospate, *AMA* anti-mitochondrial antibody, *DAA* direct –acting antivirals, *F* females, *GGT* gamma glutamyl-transferase, *HCV* Hepatitis C Virus, *IF* immunoflourescence, *LDV* ledispavir, *M* male, *ND* not detected, *Post-tx* post treatment, *RNA NAT* Ribonucleic acid quantitative nucleic acid test, *RBV* ribravin, *SOF* sofosbivur, *yrs* years, *IF* immunoflourescence

Patient 2 fulfilled diagnostic criteria for PBC on initial clinic evaluation, having both an elevated serum AP level and AMA positivity. UDCA (13 mg/kg/day) therapy was initiated but was subsequently discontinued due to intolerable side effects. The patient was not taking UDCA during the treatment period for HCV with DAA, as reflected by persistently elevated serum AP levels during the pre- and post-HCV treatment period.

Patient 3 had a positive AMA on initial investigation in the setting of a normal serum AP level, therefore failing to meet classical diagnostic criteria for PBC. Therefore, this patient was not treated with UDCA during the HCV treatment period.

**Fig. 1:**
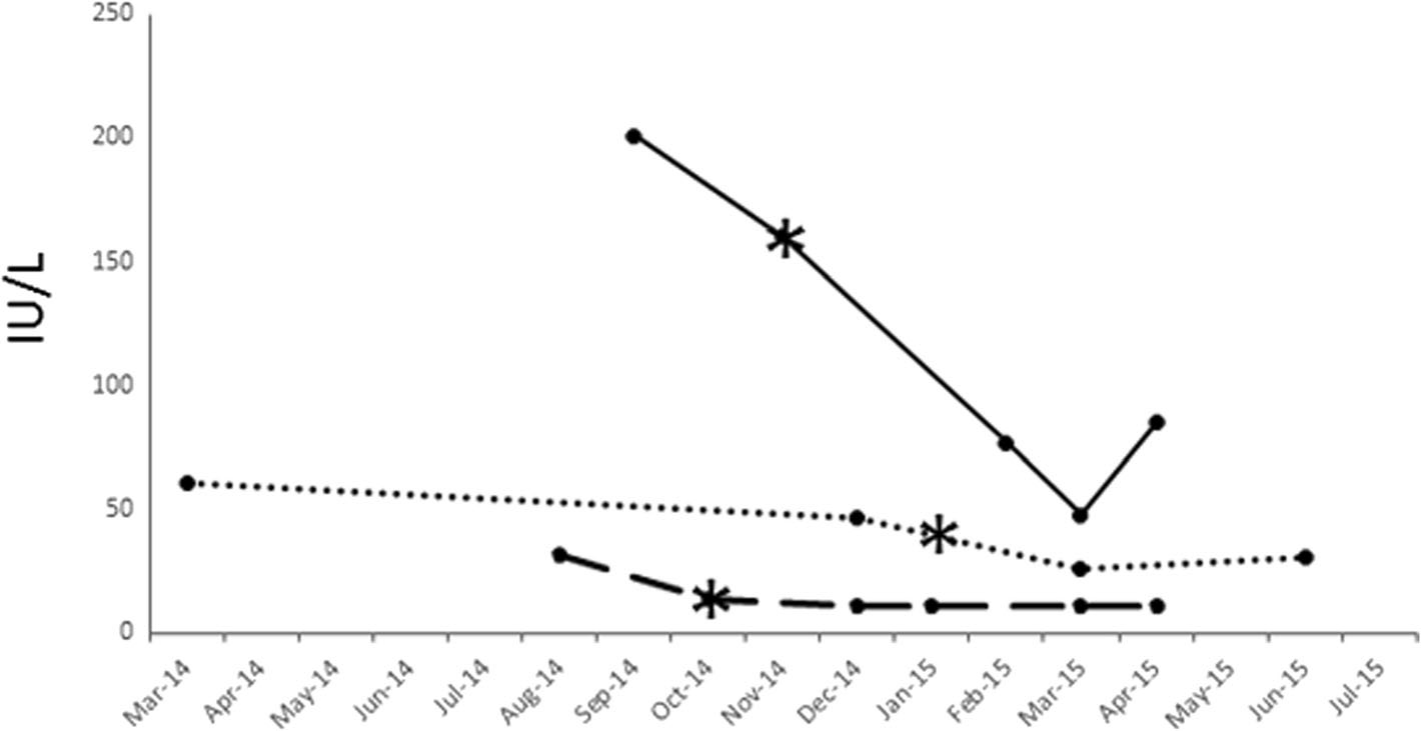
Serum alanine aminotransferase (ALT) levels of all three patients during HCV treatment. Patient 1 (solid line) was treated with ledipasvir + sofosbuvir X 12 weeks between the dates of November 29, 2014 to February 20, 2015. Patient 2 (dotted line) was treated with ledipasvir + sofosbuvir X 24 weeks between the dates of January 15, 2015 to July 2, 2015. Patient 3 (dashed line) was treated with sofosbuvir + ribavirin X 24 weeks between the dates of October 23, 2014 to April 8, 2015. Treatment initiation dates with DAA are indicated by a black asterisk (*). ALT levels were measured at different time points as shown in the X-Axis

**Fig. 2:**
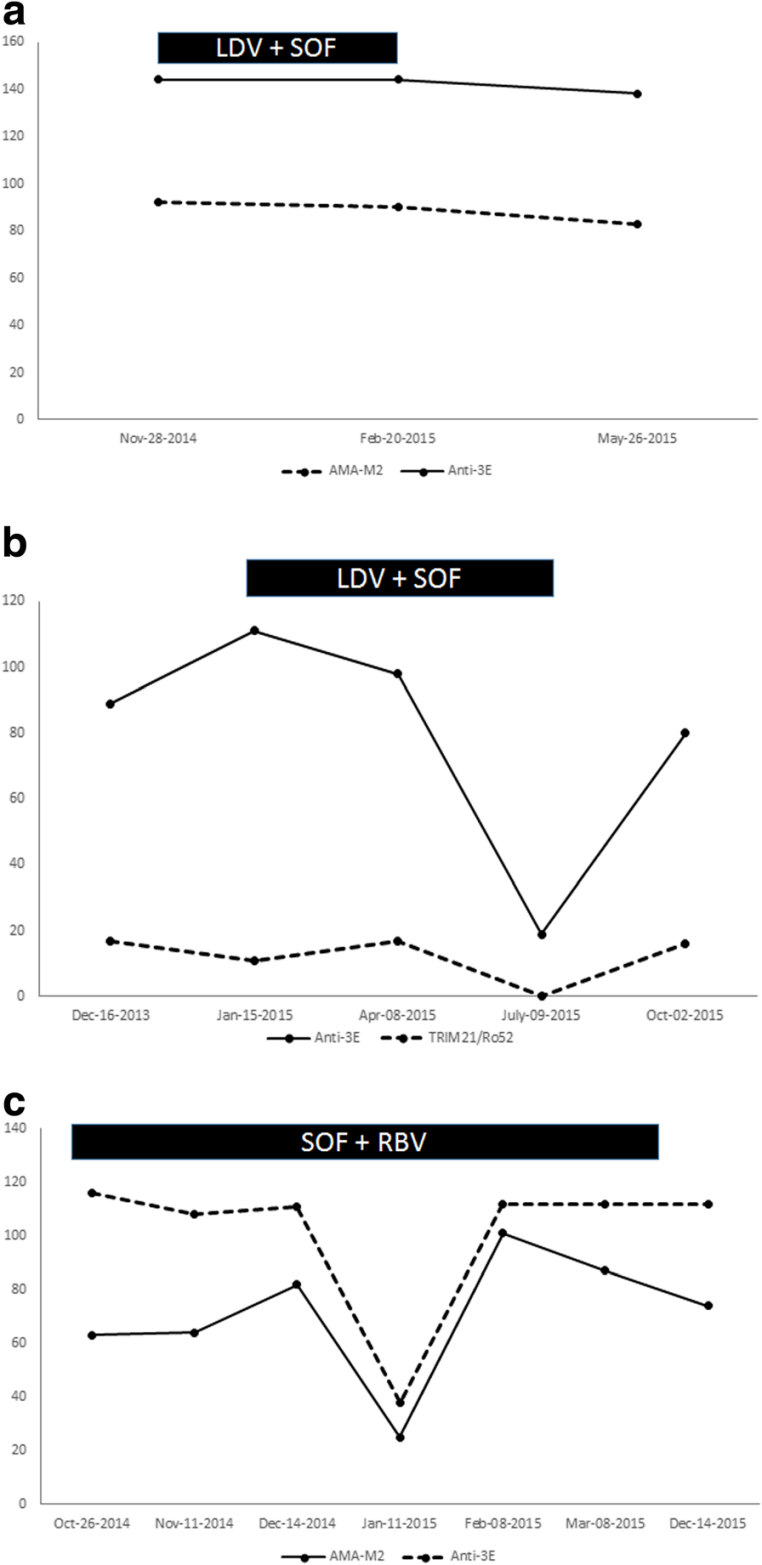
**a** Titres of anti-mitochondrial antibody M2 (AMA-M2) and anti-3E-BPO during HCV therapy with ledipasvir (LDV) and sofosbuvir (SOF) X 12 weeks in Patient 1. LDV + SOF therapy was given between the dates of November 29, 2014 to February 20, 2015 (black box). Antibody titres were measured at three different time points as shown in the X-Axis. **b** Titres of anti-TRIM21/Ro52 and anti-3E-BPO during HCV therapy with ledipasvir (LDV) + sofosbuvir (SOF) X 24 weeks in Patient 2 LDV + SOF was given between the dates January 15, 2015 to July 2, 2015 (black box). Autoantibody titres were measured at various time points as shown in the X-Axis. **c** Titres of anti-mitochondrial antibody (AMA-M2) and anti-3E-BPO during HCV therapy with Sofosbuvir (SOF) and Ribavirin (RBV) X 24 weeks in Patient 3 SOF + RBV therapy was given between the dates of October 23, 2014 to April 8, 2015 (black box). Autoantibody titres were measured at various time points as shown in the X-Axis

In all three patients serum IgG and IgM levels were within normal range on initial clinical assessment. No liver biopsies were obtained from any of the patients. All 3 patients achieved a SVR with DAA therapy. Serum alanine aminotransferase (ALT) levels demonstrated a robust early and sustained decrease in association with DAA therapy in all three patients (Fig. 1). Serum alkaline phosphatase, GGT, and total bilirubin levels remained within the normal range during DAA treatment for both Patient 1 and Patient 3 (Normal range of Alkaline Phosphatase 35–125 IU/L; GGT < 61; Bilirubin < 20 umol/L). Patient 2 had cholestatic liver enzymes with serum AP ranging from 130 to 150 IU/L and GGT 220–250 U/L during the treatment period with DAA. In 2 of 3 patients there was documented improvement in the Fibroscan® score post-HCV treatment. In one patient, a Fibroscan® assessment could not be done post-HCV treatment as the patient had left the country after finishing treatment. Serum autoantibody titres were measured for each patient before, during and after HCV treatment, and results are shown in Fig. 2. In all three patients, pre- and post-DAA treatment autoantibody titres for anti-3E-BPO, TRIM21/Ro52, and AMA (M2) were similar. In Patient 2 (Fig. 2b), the titre of anti-3E BPO exhibited a transient decrease during DAA treatment, followed by a rebound to baseline levels post-DAA therapy. This trend was also noted in Patient 3 for both AMA-M2 and anti-3E BPO titre levels (Fig. 2c).

## Discussion and conclusion

HCV was successfully eradicated by DAA treatment in all 3 of our HCV patients. However, in these successfully treated patients, serum titres of the PBC-related autoantibodies AMA (M2), anti-3E BPO and/or anti-Ro52 were similar in the pre-treatment and post-treatment serum samples. These findings suggests that ongoing HCV replication is not a significant driver of autoantibody production in PBC.

The diagnostic criteria for PBC requires both AMA positivity and concurrent elevation in serum AP levels [11]. Two patients met these diagnostic criteria, one on initial presentation (Patient 2) and the other who developed cholestatic liver enzymes in follow up (Patient 1). Only one patient did not fulfill classical diagnostic criteria for PBC; exhibiting normal serum AP levels before, during, and after HCV eradication in the setting of AMA positivity (Patient 3). Despite failure to meet diagnostic criteria, the presence of AMA at high titer (by immunofluorescence) in this patient is highly suggestive of an underlying diagnosis of PBC. AMA is a highly specific biomarker for PBC, found in up to 95% of patients, and is widely considered a hallmark autoantibody for this condition [13]. In fact, it has been shown that the majority of AMA positive patients with normal liver enzymes at baseline, will ultimately progress over time to develop overt clinical and biochemical features of PBC [14, 15]. Moreover, AMA-M2 and Anti-3E-BPO autoantibodies were also detected in this patient (Patient 3). Both of these autoantibodies have been previously described to be both prevalent in, and specific for, the diagnosis of PBC [16–18]. Taken together, this constellation of autoantibodies in Patient 3 is highly suggestive of the underlying diagnosis PBC, despite the absence of cholestatic serum biochemistry.

AMA is non-organ specific, targeting ATPase-associated antigens located on the inner mitochondrial membrane [19, 20]. There are four principal mitochondrial antigens targeted in PBC, collectively classified as the M2 subtype. These include the E2 subunits of the pyruvate dehydrogenase complex, the branched chain 2-oxo-acid dehydrogenase complex, the ketoglutaric acid dehydrogenase complex, and the dihydrolipoamide dehydrogenase-binding protein [21–23]. Despite being highly specific for PBC, AMA is one of many autoantibodies that have been documented in patients with chronic HCV infection. It is estimated that up to 8% of patients with HCV are AMA+ [4, 5, 9]. However, it is difficult to ascertain from these studies whether AMA positivity was secondary to previously undiagnosed PBC, or HCV- mediated seroconversion. AMA seroconversion has been documented in one case report of a patient with HCV, but this was in the unique setting of treatment with PEGylated interferon [24]. In our case, with 2 of 3 patients fulfilling diagnostic criteria for PBC and all three patients harboring highly specific autoantibodies for PBC, it is inferred that this is more likely a reflection of underlying PBC rather than a sequelae of seroconversion due to chronic HCV infection. However, given the established association of chronic HCV infection and autoimmune disease/ autoantibody production, larger prospective studies would be required to further define the role of HCV infection in mediating AMA seroconversion.

Contrary to its utility in diagnosing PBC, the role of AMA titers for monitoring disease activity and in defining disease prognosis in PBC appears to be of limited value [25, 26]. Anti-Ro52 is an autoantibody associated with a wide range of autoimmune conditions, including autoimmune myositis, SS, SSc, and systemic lupus erythematosus (SLE) [27]. In PBC patients, the most frequently detected extractable nuclear antigen (ENA) is that of TRIM21/Ro52 [28]. The presence of anti-TRIM21/Ro52 at the time of PBC diagnosis is associated with elevated serum IgM and bilirubin levels, and advanced liver fibrosis stage [28]. Anti-Ro52 has also been associated with increased risk of cirrhosis and hepatic death or liver transplantation in the setting of autoimmune hepatitis [29]. One of our patients had detectable serum anti-TRIM21/Ro52 before HCV treatment. Contrary to previous studies, this patient did not have an associated elevation in serum IgM or bilirubin level, despite having a positive anti-TRIM21/Ro52 level. In addition, there was no overall sustained change in the titre of anti-TRIM21/Ro52 in this patient during their successful HCV treatment.

There are however limitations to this study that warrants further discussion. In addition to the inherent biases associated with the retrospective nature of a case series, the limited sample size does not allow for generalization of our findings to all patients with HCV and PBC. A larger n-value of patients are needed to truly evaluate the effects of HCV eradication on autoimmunity/autoantibody production. Ideally, a prospectively designed study with measurements of autoimmune liver disease associated autoantibodies before and after HCV treatment are needed. This however may be challenging due the fact that patients with concurrent HCV and PBC are not common.

In conclusion, the outcome of our case series highlights that HCV eradication does not significantly impact PBC-related autoantibody production in PBC-HCV patients. The findings in our case series may suggest that HCV infection is not a significant driver of autoimmunity in PBC patients, and cannot explain previous reports suggesting more aggressive clinical disease in HCV infected PBC patients. Currently there are no therapeutic guidelines on the management of PBC-HCV patients. Our case series suggests that DAA treatment is safe and effective in this group of patients, but does not alter underlying PBC-related autoimmunity. Further studies are however needed to better elucidate the association between HCV treatment and underlying autoantibody production as well as evaluate the safety of using DAAs in patients with concurrent HCV and PBC.

## Abbreviations

3E-BPO: Branched-chain 2-oxo-acid dehydrogenase complex and 2-oxo glutarate dehydrogenase complex
AMA-M2: Antimitochondrial antibody
ANA: Antinuclear antibody
anti-LKM1: Anti-liver kidney microsome type 1
ASMA: Anti-smooth muscle antibody
DAA: Direct-acting antivirals
gp210: Glycoprotein 210
HCV NAT: Hepatitis C Virus Quantitative Nucleic Acid Test
HCV: Hepatitis C virus
LC-1: Liver cytosolic-1 antigen
PBC: Primary Biliary Cholangitis
PML: Promyelocytic leukemia cell antigen
SLA: Soluble liver antigen
sp100: sp100 nuclear antigen
SS: Sjogren’s syndrome
SSc: Systemic sclerosis
TRIM21/Ro52: Tripartite motif-containing protein 21
